# Self-Consistent Soundscape Ranking Index: The Case of an Urban Park

**DOI:** 10.3390/s23073401

**Published:** 2023-03-23

**Authors:** Roberto Benocci, Andrea Afify, Andrea Potenza, H. Eduardo Roman, Giovanni Zambon

**Affiliations:** 1 Department of Earth and Environmental Sciences (DISAT), University of Milano-Bicocca, Piazza della Scienza 1, 20126 Milano, Italy; 2 Department of Physics, University of Milano-Bicocca, Piazza della Scienza 3, 20126 Milano, Italy

**Keywords:** soundscape, soundscape ranking index (SRI), urban parks, acoustic sensor networks

## Abstract

We have performed a detailed analysis of the soundscape inside an urban park (located in the city of Milan) based on simultaneous sound recordings at 16 locations within the park. The sound sensors were deployed over a regular grid covering an area of about 22 hectares, surrounded by a variety of anthropophonic sources. The recordings span 3.5 h each over a period of four consecutive days. We aimed at determining a soundscape ranking index (SRI) evaluated at each site in the grid by introducing 4 unknown parameters. To this end, a careful aural survey from a single day was performed in order to identify the presence of 19 predefined sound categories within a minute, every 3 minutes of recording. It is found that all SRI values fluctuate considerably within the 70 time intervals considered. The corresponding histograms were used to define a dissimilarity function for each pair of sites. Dissimilarity was found to increase significantly with the inter-site distance in space. Optimal values of the 4 parameters were obtained by minimizing the standard deviation of the data, consistent with a fifth parameter describing the variation of dissimilarity with distance. As a result, we classify the sites into three main categories: “poor”, “medium” and “good” environmental sound quality. This study can be useful to assess the quality of a soundscape in general situations.

## 1. Introduction

The acoustic quality of a habitat is generally recognized as a primordial requirement of wildlife conservation [1,2]. It has become customary to evaluate the environmental status of particularly exposed areas, such as large populated urban zones, in addition to natural habitats. As a result of increasing urbanization all over the world, the associated, and in many cases excessive human technophonies, usually called “noise”, together with a lack of planning to permit for a smooth transition between a built environment and a natural one, can yield several deleterious effects on biodiversity. An exhaustive picture on these issues has been documented in recents works [3,4,5,6].

The use of passive acoustic monitoring has become the main tool to study a particular habitat [7,8,9,10]. Its diffusion among researchers and technicians has evolved considerably together with its increasing memory capability. An important step towards its utilization on large scales came with the development of specific spectral and level characteristics of sound through the analysis of ecoacoustic indices (for a review, see [11]). This, in addition, allows the retrieval of important information about the way different sounds are assembled across both space and time. This new characteristic of the habitat sound is commonly referred to as the soundscape [12,13], and it has been recognized as a distinct feature or, more importantly, as an ecological “signature” of a landscape [14,15,16].

Urban parks are sources of natural sounds, but in many cases, they are mixed with anthropogenic noise, such as vehicle traffic noise, from surrounding built areas [17,18]. Many research works have shown that Brazilian and Italian parks exceed noise level thresholds due to the presence of traffic noise as the main source of disturbance [19,20,21]. These results also give evidence that the traffic noise perception in urban parks can undermine the potential beneficial effects of natural sounds [22].

The ecoacoustics indices merge the complex acoustic dynamics of an ecosystem, consisting of vocalizing species, anthropogenic noise, and natural phenomena [23], into sets of time series, allowing us to get a picture of the environmental changes at a given habitat [24]. Furthermore, they provide new insights on species diversity and human impacts across a wide range of terrestrial [25,26,27,28,29] and aquatic environments [30,31]. The validation of the calculation of ecoacoustic indices is usually sound-truthed by specialized operators who classify hours of recordings according to predefined sound categories.

The identification of sound sources by operators is highly time consuming and requires specific knowledge of animal vocalizations, thus limiting its applicability to small datasets [32,33]. However, a cumulative approach based on a qualitative description of the recorded sound (e.g., many/few vocalizing birds, many/few birds species, high/low traffic noise, etc.) was shown to improve the validation process, showing a good matching between the dentification of acoustic categories from the audio recording and the ecoacoustic indices [34].

In this work, we address the question of quantifying the quality of a local soundscape from a set of audio recordings at each site of a grid covering the area of interest. The intervention of an expert is required, who is expected to recognize the different sound categories determining the soundscape. The main goal is to associate a soundscape ranking index (SRI) with each site of the grid, in order to classify the quality of the local environmental sound into one of the following three categories: “poor”, “medium” and “good”. This is achieved by introducing a sound dissimilarity function between sites, found to be consistent with a power-law behavior with the inter-site distance. We conclude with a discussion of possible future work along these lines.

## 2. Materials and Methods

### 2.1. Area of the Study

**Figure 1:**
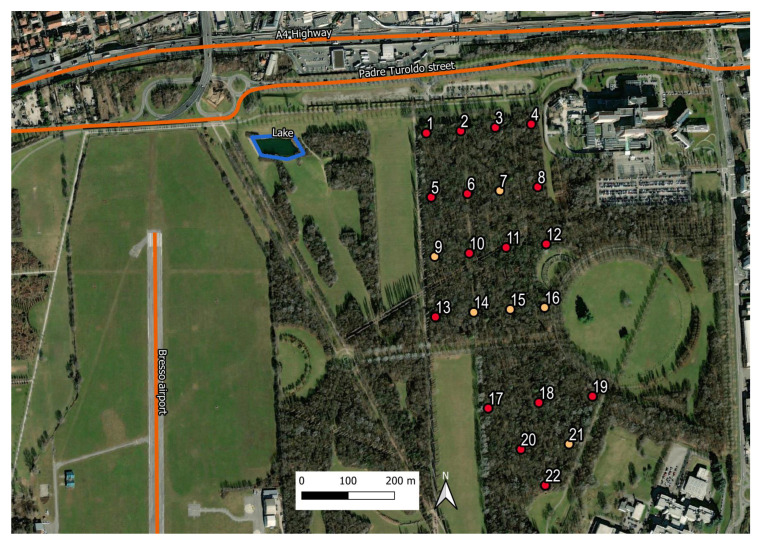
The Parco Nord (Northern Park) area of study. The sensor network is indicated by the numbered dots (1–22), deployed inside the tree-covered zone of the park. The network consists of two regular grids, the northern and the southern ones, composed of 16 and 6 nodes, respectively. Those in red color are the actually used sensors (total number 16), while those in yellow are the six ones discarded due to malfunctioning. One can see the A4 highway, the Padre Turoldo street and the artificial lake to the northern part of the park, while to the west side of it, the small Bresso airport runaway is located.

The Parco Nord (Northern Park) of Milan (Italy) covers an area of approximately 790 hectares and is located within a highly urbanized area (see Figure 1). About 45% of its surface is dedicated to natural green spots and vegetation, while the remaining parts are devoted to agricultural activities and infrastructures. The area of study is a tree-covered parcel of approximately 22 hectares enclosed by agricultural fields, lawns, paths and roads. It has a semi-natural structure, which is characterized by herbaceous layers of nemoral flora, shrub and arboreal layers and dead woods.

The zone is crossed by numerous paths, and it is mainly used for recreational activities. It contains an artificial lake of about 300 m${}^{2}$ surface area, located at approximately 250 m from the edge of the bush. The main traffic noise sources are the A4 highway and the Padre Turoldo street, both located north of the park at around 100 m from the wooded parcel. There is also the presence of a small airport (Bresso airport) on the west side at around 500 m from the tree-line edge.

### 2.2. Audio Recorders

Mapping the environmental sounds over large areas may require important financial commitments that could be alleviated by the availability of very low-cost recorders (VLCRs). Thus, we used SMT security digital audio recorders with a 48 kHz sampling rate and equipped with a two-week lifetime powerbank. The main disadvantage of using very low-cost recorders is the possibility that they present dissimilar microphone sensitivities. This may cause a different frequency and level response to a sound exposure. The response of each recorder was evaluated in terms of the following:

- Computation of the acoustic complexity index (ACI, see [27]) for a white noise source.
- Behavior of each VLCR at different frequencies.

The ACI computes the relative variation of recorded amplitudes of adjacent temporal steps in each frequency bin, as determined by the FFT analysis in the frequency range 0–24 kHz. Thus, its computation provides overall evidence of possible anomalies in the frequency response. Anomalies were identified when the recorder response strongly departed from the average ACI value. Based on these results, we selected audio recorders characterized by a response within 3% of the average ACI value. The full description of the procedure is reported in [35].

### 2.3. Measurement Scheme

The 22 recorders were initially positioned on two regular grids (see Figure 1), the first one (northen part) covering an area of approximately 500 × 270 m${}^{2}$, and the second grid (southern part) covering an area of about 300 × 270 m${}^{2}$. The recordings were scheduled for the period of greatest singing activity of the avifauna [10] and repeated over four days, namely on 25–28 May 2015, from 06:30 a.m. (UTC +2) to 10:00 a.m., corresponding to a 3.5 h long recording session for each day. Unfortunately, six recorders, numbered as 7, 9, 14, 15, 16 and 21 and indicated by the yellow spots in Figure 1, did not work properly, and thus, the audio files analyzed in this study reduced to only 16 sites.

### 2.4. Aural Survey

In order to quantify the biophonies [36], anthrophonies and geophonies [37], an aural survey was performed on one of the four days of recordings, of 3.5 h total duration for each of the 16 sites. Specifically, a single expert listened carefully to the 16 recordings according to the following scheme. For each site, the daily record duration of 210 min was grouped into 70 intervals of 3 min each, of which only the first 1-min interval was listened, while the following 2 min of recording were skipped. This operation spanned several weeks of careful work to accurately examine the whole set of recordings. The expert focused on quantifying the biophonic activity (mainly avian vocalizations) and technophonic sources (mainly traffic noise, including trains and airplanes, and other sources such as park maintenance activities), according to the scheme discussed in [38] and reported in detail in Table 1.

For each single minute of listening, bird vocalizations and non-biophonic sources were searched. The former was subdivided into three main categories according to the quantity of birds (many, few, none), number of species (>1, 1, none) and sound durations, (100, 75, 50, 25, 0)%. The third column displays the index *n* of the parameter P(n) (n=1,5), associated with each subcategory (i=1,19). The fourth column is aimed at providing a more specific characterization of the way the subcategory could be identified. Regarding the bird sound durations, the latter is expressed in terms of the percentage of bird singing activity identified within the considered minute. The effective time span in seconds is displayed in the fourth column.

The non-biophonic contributions are split into road traffic, trains, airplanes and other technophonic noise sources. The former is subdivided into two categories, level and type of traffic, while the last three are associated with the fifth parameter if they are present and with the third one if absent (see below). Finally, geophonies such as rain and wind were not considered due to their negligible contribution to the soundscape during the measurement campaign.

### 2.5. The Soundscape Ranking Index

We are interested in quantifying the quality of the local environment sound by means of a soundscape ranking index, SRI, as proposed recently [38]. In the following, we briefly discuss how to evaluate the SRI, aimed at describing the local soundscape, at a given site *j* in the network, in an average sense. The value of SRI(j,t) depends on the time interval *t*, in our case (t=1,70), listened during the aural survey at site *j*. For each time interval *t* and site *j*, we determine the event function, N(i,j,t) for the *i*th sound category (i=1,19), described in Table 1. The event function can be either N=1 or N=0, depending on whether the event *i* is present or not. The SRI can then be obtained as the sum

(1) $$\mathrm{SRI}\left(j,t\right)=\sum_{i=1}^{19}N\left(i,j,t\right)\;w\left(i\right),$$

where the weights, $w\left(i\right)=P\left(n_{i}\right)\;c\left(n_{i}\right)$, with $n_{i}=\left(1,\;2,\;3,\;1,\;2,\;3,\;1,\;1,\;1,\;1,\;1,\;2,\;4,\;5,\;2,\;4,\;5,5,\;5\right)$ (cf. Table 1), while the additional factor, $c\left(n_{i}\right)=\left(1.0,\;0.75,\;0.50,\;0.25,\;0.0\right)$ for i=(7,8,9,10,11), respectively, and $c(n_{i})\;=\;1$, otherwise. For instance, w(6)=P(3)c(6)=P(3), while w(7)=P(1)c(7)=0, and w(8)=P(1)0.25, etc.

The choice of the values for the parameter P(n) is rather arbitrary of course, but we can stick to the following simple considerations in order to make up a representative and useful picture of the soundscape. We follow our previous work [38] and assume that P(n)>0 if the sound is associated with a natural source, while we take P(n)<0 if it is of anthropogenic origin. Given the fact that we consider five different situations, we chose the values reported in Table 2 to start with.

Now, we have all the ingredients to calculate the SRI(j,t), using Equation (1), once the event function, N(i,j,t), is known for all the subcategories *i*, at site *j* and time interval *t*. SRI is expected to fluctuate as a function of *t*, reflecting the ever changing environmental conditions of the varying soundscape. However, we may average the index over time in order to get a mean value, 〈SRI(j)〉, at site *j*, which should provide us with a quantitative element to estimate the *quality* of the local environmental sound. By quality, we mean that 〈SRI(j)〉 should be large for a natural soundscape and small for a poor one affected by strong anthropogenic perturbations. At this point, and in order to fix the ideas, we suggest a simple classification of the mean quality index, 〈SRI〉, as displayed in Table 3.

### 2.6. Optimization Procedure for P(n)

The values of P(n) reported in Table 2 can be seen as our starting guess of the unknown parameters and are therefore arbitrary. As we discuss in Section 3, however, they appear to build a quite robust starting set from which one can search for new “optimized” values. In addition, we find that the optimized set is closer to the prescription in Table 3 than the original set. We show that this different behavior is a result of the self-consistent optimization method we have developed for obtaining the parameters P(n).

The basic quantity in our approach is the probability distribution function, $H_{j}\left(\mathrm{SRI}\right)$, of the set of SRI values obtained at a given site *j*, at different time intervals *t*, using Equation (1). Based on the distribution functions obtained for all active sites, we can define a quantity representing how “dissimilar” two distributions are. The dissimilarity between say, sites *i* and *j*, denoted as $D_{i,j}$, is here defined by the following relation:

(2) $$D_{i,j}=1-\frac{1}{A_{i}\;A_{j}}\int_{-\infty }^{\infty }dx\;\;H_{i}\left(x\right)\;H_{j}\left(x\right),\;\;\;\;\mathrm{with}\;\;\;\;\;\;A_{i}^{2}=\int_{-\infty }^{\infty }dx\;\;H_{i}^{2}\left(x\right),$$

where the normalizing factor $A_{i}$ ensures that $D_{i,i}=0$. The integral form used in Equation (2) can be formally seen as the internal product of two vectors, $H_{i}\left(m\right)$ and $H_{j}\left(m\right)$, where m∈Z are the coordinates in a high-dimensional space. Specifically, if we define the histograms $H_{i}\left(x\right)$ on a set of discrete coordinates, x=mb, where *b* is the bin size used to build the histograms, we may regard $H_{i}\left(m\right)$ as the *m*th component of the vector ${\bar{H}}_{i}$. This internal product is reminiscent of a similar form, typically used in financial studies, to define a distance between time series in terms of their cross-correlations [39]. Intuitively, the larger $D_{i,j}$ is, the larger the soundscape dissimilarity between sites *i* and *j*. In other words, Equation (2) represents a measure that can be used to estimate a soundscape distance between sites. We can loosely say that two sites displaying very different SRI distribution functions are very distant in soundscape space.

Actually, sites *i* and *j* are located at well defined positions in space; in particular, they are at a fixed spatial distance, $R_{i,j}$, within the network (cf. Figure 1). Therefore, the question arises of whether both distances, $D_{i,j}$ and $R_{i,j}$, are somehow related to each other. Intuitively, one would expect that dissimilarity increases with distance. In other words, we are interested in finding how the local soundscape changes as a function of spatial distance *R* within the network. To do this, we assume, as our working hypothesis, a simple relation of the form

(3) $$D_{i,j}\;≃\;a\;\;{\left(R_{i,j}\left[m\right]\right)}^{\alpha },$$

where the distances $R_{i,j}$ are expressed in meters, and the exponent α>0 needs to be determined empirically. We expect that Equation (3) should be valid at least in an average sense. Note that it can be written as $D∼{\left(R/R_{0}\right)}^{\alpha }$, where $R_{0}$ is an effective length scale associated with the network soundscape. This in keeping with the fact that *D* is actually dimensionless. Numerically, the constants *a* and $R_{0}$ are related to each other by, $R_{0}=a^{-1/\alpha }$. Note that $R_{0}$ is given in meters (see also Equation (4)). To simplify the notation, in what follows, we simply write $R_{i,j}^{\alpha }$, meaning ${\left(R_{i,j}\left[m\right]\right)}^{\alpha }$.

As a matter of fact, α becomes the sixth parameter in our approach. Notice, however, that P(3)=0 in all cases, so we are effectively dealing with only five parameters: P(1), P(2), P(4), P(5) and α. The five parameters are obtained by a least-square fit that minimizes the total deviation of the data from the fit, i.e.,

(4) $$\Sigma^{2}=\frac{1}{120}\sum_{i=1,j>i}^{16}{\left(D_{i,j}-a\;\;R_{i,j}^{\alpha }\right)}^{2},\;\;\;\;\mathrm{with}\;\;\;\;\;\;a=\langle D_{i,j}R_{i,j}^{\alpha }\rangle /\langle R_{i,j}^{2\alpha }\rangle \equiv R_{0}^{-\alpha }.$$

The constant *a* is obtained by requiring ∂Σ/∂a=0, and as a result, it is a function of all the five unknown parameters. We note that in the expression for *a*, the symbols represent averages over the 120 distinct site pairs (i,j) in the network. The effective soundscape length $R_{0}$ now becomes

(5) $$R_{0}={\left[\langle R_{i,j}^{2\alpha }\rangle /\langle D_{i,j}R_{i,j}^{\alpha }\rangle \right]}^{1/\alpha }.$$

## 3. Results

The results presented in this section refer to the audio files recorded on 25 May 2015, from 06:30 a.m. to 10:00 a.m., and based on the aural survey discussed in Section 2.4.

### 3.1. Initial P(N) Values

**Figure 2:**
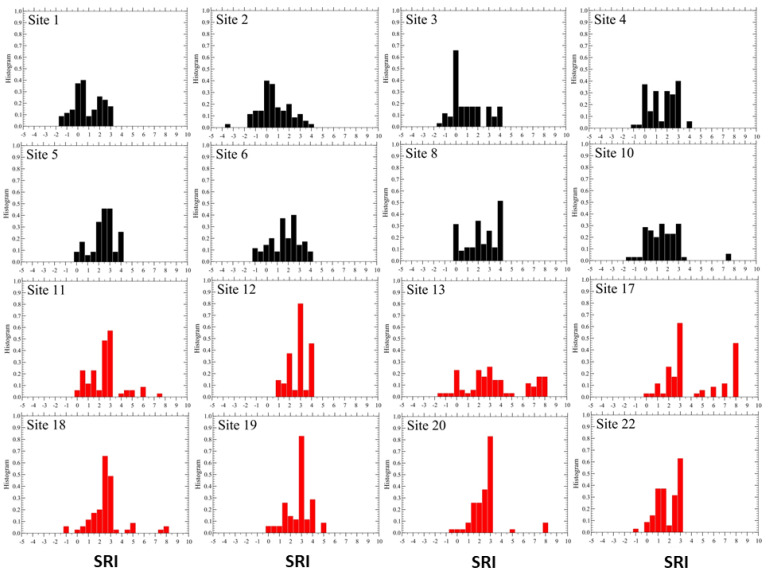
Variability of SRI at each active site. The distribution functions *H*(SRI) were obtained using Equation (1), from the initial values of parameters P(n)=(2,1,0,−1,−2). The scale for SRI is fixed for all sites in the range (−5, 10). The two sets of colors (black on top and red on bottom panel) are for convenience but indicate that the former are centered to lower values of SRI while the latter are centered to more positive ones, suggesting an underlying organization of the sites into two main groups.

We start the analysis of the soundscape using the initial values of the parameters reported in Table 2. As discussed in Section 2.5, we evaluate the SRI values using Equation (1) at the 70 time intervals for each of the 16 active sites. The corresponding histograms vs. SRI are reported in Figure 2.

**Figure 3:**
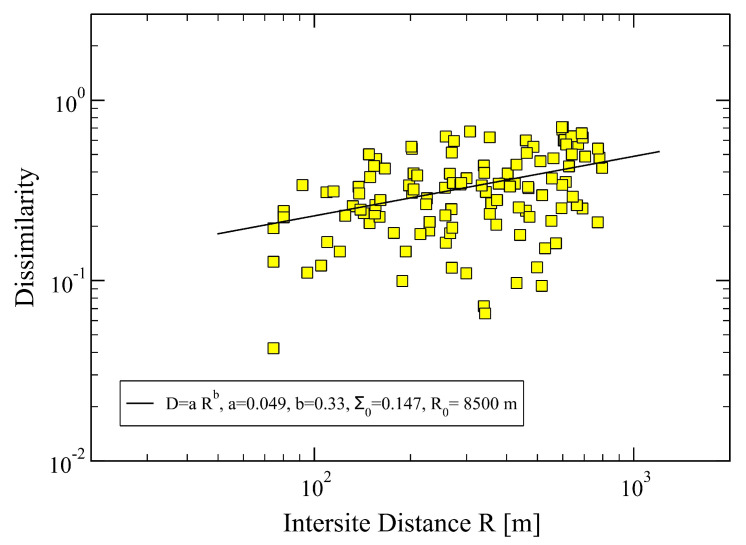
Dissimilarity, $D_{i,j}$, between two sites, (i,j) vs. inter-site distance $R_{i,j}$, obtained from Equation (2) using the initial set of parameters P(n)=(2,1,0,−1,−2). The full squares are the empirical data, and the straight line is a least-square fit with the power law $D=aR^{\alpha }$, yielding a=0.049, corresponding to $R_{0}=8500$ m, and α≃1/3. Specifically, we find $\langle DR^{\alpha }\rangle ≃2.354$ and $\langle R^{2\alpha }\rangle ≃48.03$. The total error of the fit is $\Sigma_{0}=0.147$. The mean inter-site distance in the network is $\langle R_{i,j}\rangle =353.64$ m, the minimum and maximum ones: $R_{\min}=75$ m and $R_{\max}=800$ m, respectively.

Once the distribution functions, *H*(SRI), for each active site have been obtained (Figure 2), we can evaluate, using Equation (2), the dissimilarity distances between pairs of sites. We note that, so far, the exponent α has not been required. Indeed, it can be read off from the plot of $D_{i,j}$ values versus the corresponding inter-site distances $R_{i,j}$, as shown in Figure 3. We notice in the figure a pronounced scattering of the empirical data from the behavior expected from Equation (3). Despite this fact, we can still recognize an increasing trend of *D* vs. *R*, as visualized by the obtained least-square fit representing the expected power-law featured in Equation (3). From the latter, we find α≃1/3, which is rather small, but significant enough to conclude that the assumed power-law (Equation (3)) may be acceptable.

**Figure 4:**
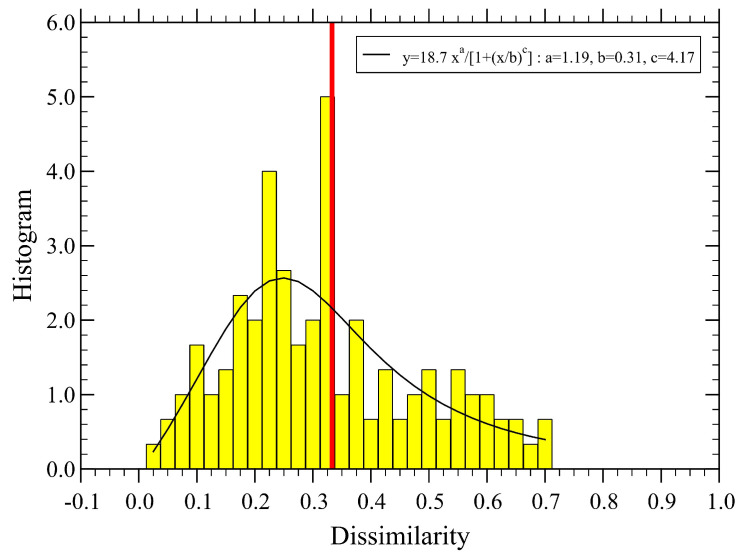
Probability density distribution of dissimilarity, corresponding to the values used in Figure 3. We find $\langle D\rangle ≃0.333$ (vertical red line). The continuous line is a fit with the form $y=Ax^{a}/\left[1+{\left(x/b\right)}^{c}\right]$, with the normalization constant A=19, and the fit parameters a=1.19, b=0.31 and c=4.17.

The associated distribution function of dissimilarity is shown in Figure 4, together with a fitting function of a simple analytical form, which should be useful for making a quantitative comparison with the case of optimized parameters discussed below.

**Figure 5:**
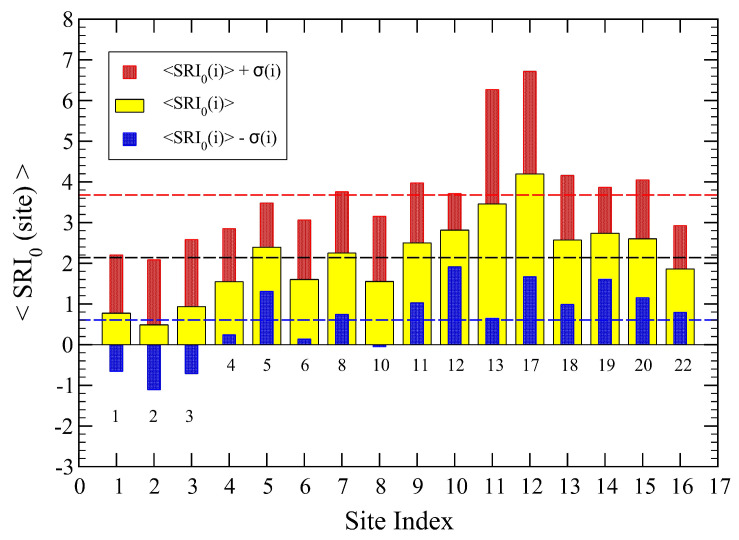
Mean 〈SRI${}_{0}$〉 obtained by averaging the index over the 70 temporal values for each active site (yellow bars). The red and blue bars display the one σ(i) variations of the data at each site *i*, above and below the local mean values, respectively. The dashed lines represent averages over all sites of the three set of data. We find 〈SRI${}_{0}$〉 = 2.14, 〈SRI${}_{0}$〉+σ = 3.68, 〈SRI${}_{0}$〉−σ = 0.60, with σ=1.54. The actual site identification numbers are reported just below the zero line: (1–6, 8, 10–13, 17–20, 22).

Finally, we plot in Figure 5 the mean SRI values at each site, obtained from Equation (1) and corresponding to the parameter set: P(n)=(2,1,0,−1,−2) and α=0.33. We note that all mean values turn out to be positive, 〈SRI〉 >0, and they are not quite consistent with the scenario expected from Table 3. This observation actually motivated us to search for a way to improve on this quality scenario results. The idea is then to search for a “better” set of parameters that can bring us beyond our initial guess, thus eliminating the arbitrariness endowed in our parameter values.

### 3.2. Optimized P(N) Values

In order to improve on the previous results, we let all the five parameters, P(1), P(2), P(4), P(5) and α, vary independently and search for the “best” set that minimizes the function Σ in Equation (4). For each set of new parameters, we need to evaluate the SRI given by Equation (1), followed by the calculation of the dissimilarity distances, Equation (2), once the corresponding histograms of the SRI have been obtained. To speed up the search, we start varying each parameter value within a given range, subdivided into few equidistant smaller intervals. Once a minimum has been found, we choose new ranges centered around the putative minimum parameter values. In this way, we can refine the search more efficiently and converge fast towards a solution. Indeed, few such iterations are needed to get a stable result.

It is convenient to check the convergence of the method by first keeping α fixed and letting the P(n) values adjust themselves so as to minimize Σ. We tried both, α=0.33 and α=1, and from each of the found sets, we minimized Σ with respect to α. We obtained similar values for α in both cases, yielding α≈0.5±0.2. We then performed a full search with all five parameters, finding indeed α≃0.5±0.05, suggesting that we may take α=1/2 as our best value for this unknown exponent. Finally, we set α=1/2 and optimized the search for our final set of parameters, yielding

**Figure 6:**
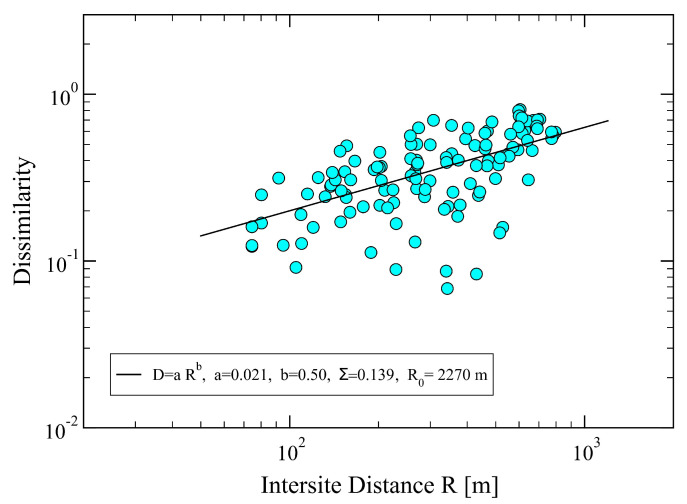
Dissimilarity, $D_{i,j}$, between two sites, (i,j) vs. inter-site distance $R_{i,j}$, obtained from the optimized parameters P(n)=(2.29,0.766,0,−1.528,−2.262), corresponding to the best value α=1/2. The full circles are the empirical data, and the straight line is a least-square fit with the power law $D=aR^{\alpha }$, yielding a=0.021, corresponding to $R_{0}=2270$ m, with $\langle DR^{\alpha }\rangle ≃7.39$ and $\langle R^{2\alpha }\rangle ≃353.6$. The total error of the fit is Σ=0.139, which is smaller than the one obtained for the initial parameter values, $\Sigma_{0}=0.147$ (cf. Figure 3).

(6) P(1)=2.290,P(2)=0.766,P(4)=−1.528,P(5)=−2.262,

with P(3)=0, and a final error, Σ = 0.139, for dissimilarity versus inter-site distance, as shown in Figure 6.

**Figure 7:**
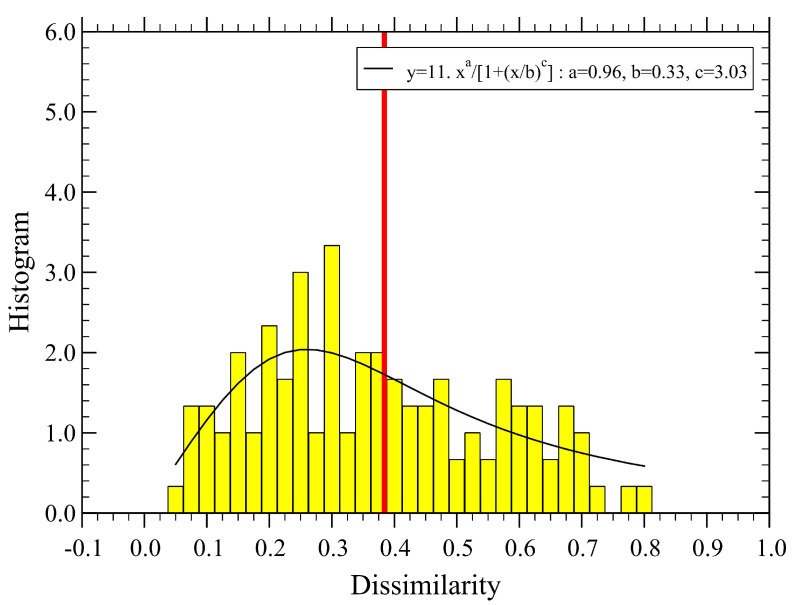
Probability density distribution of dissimilarity, corresponding to the values used in Figure 6. We find $\langle D\rangle ≃0.375$ (vertical red line). The continuous line is a fit with the form $y=Ax^{a}/\left[1+{\left(x/b\right)}^{c}\right]$, with the normalization constant A=11 and the fit parameters a=0.96, b=0.33 and c=3.03.

The distribution function of the newly obtained dissimilarity distances, $D_{i,j}$, is displayed in Figure 7. Now, the mean dissimilarity, $\langle D\rangle ≃0.375$, becomes a bit larger than for the non-optimized parameters (cf. Figure 4), while the new distribution function becomes flatter and extends to larger values of *D*.

**Figure 8:**
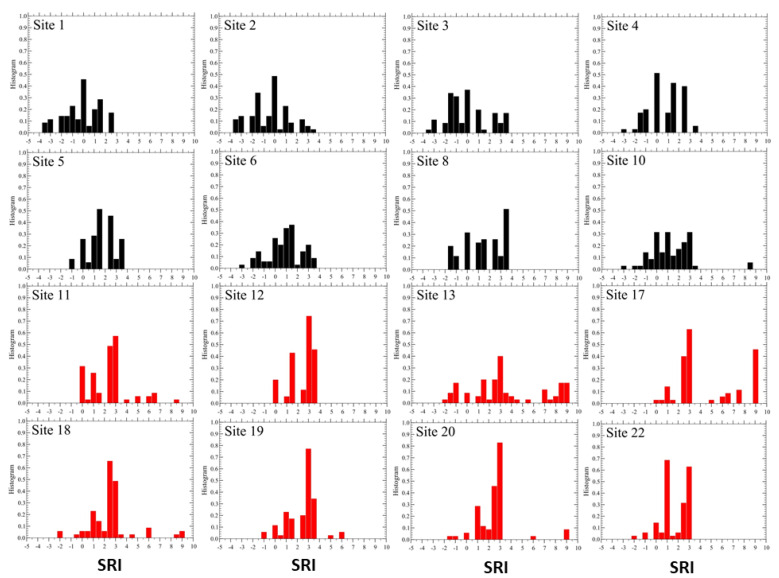
The distribution functions *H*(SRI) for the optimized parameters displayed in Equation (6). The scale for SRI is the same used in Figure 2. Here again, we have used two sets of colors for the histograms.

**Figure 9:**
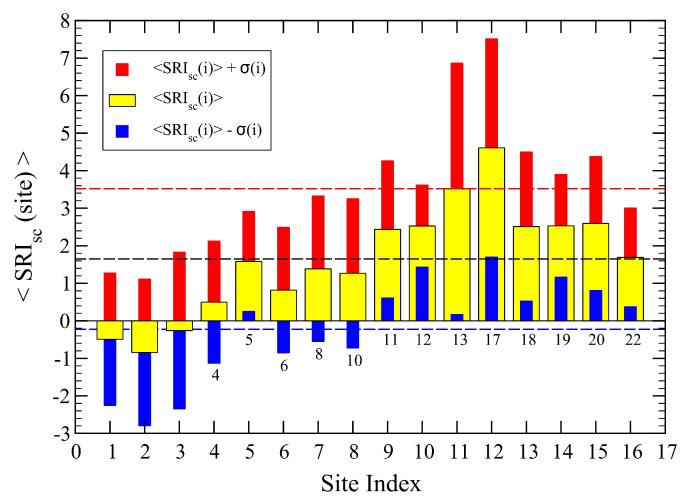
Same as in Figure 5 for the self-consistent set of parameters (Equation (6)). Here, we find 〈SRI〉 = 1.65 (2.14), 〈SRI〉+σ = 3.53 (3.68), 〈SRI〉-σ = −0.23 (0.60), with σ=1.88 (1.54). The corresponding values from Figure 5 are reported in parenthesis.

The distribution functions of the SRI for each site are displayed in Figure 8, and their mean values are shown in Figure 9.

## 4. Discussion and Conclusions

It is convenient to summarize the main results of our work as shown in Table 4. Let us start with the exponent α, expressing the way that dissimilarity decays with inter-site distance (Equation (3)). Quantitatively, the values indicate that for the initial set of parameters, dissimilarity decays more slowly (1/3) than for the optimized one (1/2), suggesting a more persistent behavior of the soundscape. The optimized values for the parameters P(n) display the same trend as the initial ones, differing by at most a 50 % as for P(4). Despite this, crucial differences develop when we analyze the remaining quantities.

The SRI behaves quite differently. Indeed, the optimized set yields a much smaller mean SRI value for the whole network, about 23 %, than for the original non-optimized set of parameters. This again is consistent with the behavior of dissimilarity, indicating a lower soundscape quality than the one predicted by the original set. The dispersion of the SRI at each site, σ(i), increases for the optimized parameter set, consistent with a more extended variability of dissimilarity (cf. Figure 4 and Figure 7), also reflected in their mean values, $\langle D\rangle$. The effective length scale $R_{0}$ also manifests this difference as it is larger for the original set, suggesting that dissimilarity is smaller than for the case of optimized parameters. Finally, as expected, the optimized set yields a smaller dispersion of the data from the assumed power-law decay of dissimilarity as given by its smaller value of Σ.

We may conclude that in our case, the initial guess for the parameter values overestimate the sound quality in the urban habitat we studied. This conclusion is based on the smaller dispersion of the data obtained from the assumed empirical relation between dissimilarity and intersite distance, as shown in Equation (3). We may expect that in other habitats, the self-consistent parameters would be different from the initially chosen values in either way, i.e., the soundscape quality may be either smaller or larger than initially predicted. In both cases, the present method suggests which of the scenarios is more likely to be correct, as it provides us with a recipe to check for the self-consistency of the empirical data. From a more fundamental perspective, the problem of deriving the basic relation in Equation (3) should be considered for future studies.

In view of these results, we may suggest that sound dissimilarity between sites decays with their inter-site distance in space with an exponent α=1/2, yielding

(7) $$D_{i,j}\;≃\;{\left[R_{i,j}/R_{0}\right]}^{1/2},$$

where the effective length scale in the network is given by,

(8) $$R_{0}={\langle R_{i,j}\rangle }^{2}/{\langle D_{i,j}\sqrt{R_{i,j}}\rangle }^{2}.$$

These relations can be tested in other cases, and if confirmed, they may represent a useful technique to estimate the internal correlations of the soundscape in the area of interest. Indeed, the set of parameters P(n), obtained in a self-consistent fashion with the assumed decay of dissimilarity, allows us to classify the sites according to our scheme discussed in Table 3.

**Figure 10:**
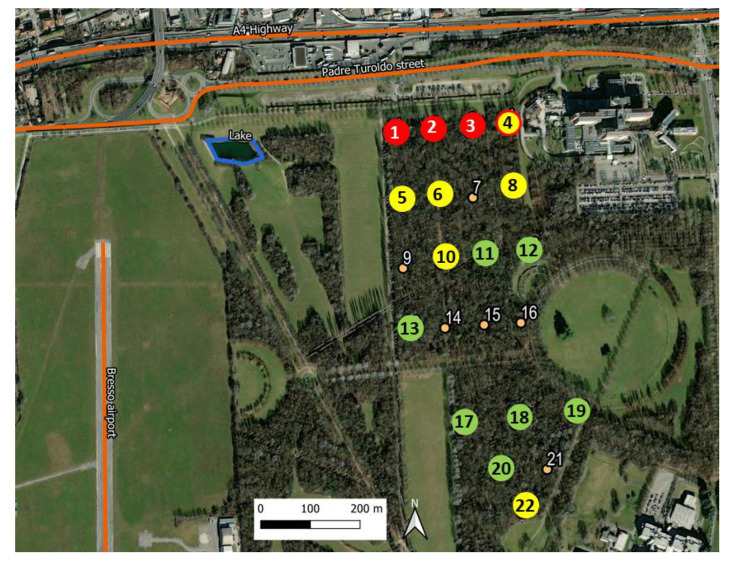
Final classification of the active sensors: (red circles) 〈SRI〉<0, (yellow circles) 0<〈 SRI 〉<2 (sensor 4 has been indicated with a red border line since its SRI≃0), and (green circles) 〈SRI〉>2. The latter represents the best quality locations in the network. The value of the optimized parameters are displayed in Table 4. The inactive sensors (7, 9, 14, 15, 16, 21) are depicted by the small circles.

According to the results in Figure 9, we find it quite remarkable that the local SRI mean values are consistent with our simple quality rules displayed in Table 3. We have therefore classified them according to these rules and plotted the corresponding set of sites with different colors, as shown in Figure 10. The good quality sites are in green color, the medium quality ones in yellow and the poor quality sites in red. There is a “borderline” site, number 4, which has a mean SRI close to 0. We have therefore indicated it as a full yellow circle with a red contour.

Environmental sound represents an important phenomenon that is fully integrated in the ecological system as a whole [40]. This feature is strongly interconnected with the ecological processes and patterns driven by biotic and abiotic relationships [40,41]. For this reason, environmental sounds may represent measurable indicators of ecological relationships and environmental degradation [42,43]. In addition, a simple but effective classification scheme to visualize the internal “structure” of the soundscape inside a habitat should be useful for many applications, including helping the monitoring of a given area for implementing better environmental policies.

One may also attempt to apply pattern recognition algorithms to analyze larger audio recorders in a systematic way, in order to extract the sound subcategories (and possibly other ones) considered in Table 1. This could be of great help, and one can rely on human intervention only to validate the fully automatized algorithm. Our approach represents a methodological effort to describe the environmental sound quality on the basis of a receiver perception. For this reason, although we used short recording periods (3.5 h), obtained from an area of approximately 22 hectares, and some of the geophonic categories were not represented in the audio recordings (wind, rain), we expect this study to provide a useful measure of a habitat soundscape quality in terms of a simple SRI index.

Finally, we expect these results to be useful also in connection with more elaborated approaches based on ecoacoustic indices and associated time series, analyzed using artificial intelligence (AI) methods, as they may serve as simpler qualitative indicators of the soundscape quality to complement the AI results. Work in this direction is in progress.

## Figures and Tables

**Table 1:** Sound categories corresponding to the 19 identified sources. (First column) Sound sources. (Second column) Quantity/Duration/Level. (Third column) Parameter index $n_{i}$ ($n_{i}\in \left\{1-5\right\}$ and i=1,19) associated with the *i*th category. (Fourth column) Identification feature.

Sound Source	Quantity	Parameters	Identification
	Many	1	Many birds
Birds number	Few	2	Few birds, no traffic
	None	3	No birds, no other sources
	>1	1	Many species
Birds species	1	2	One species, no traffic
	None	3	No birds, no other sources
	100%	1	60 s
Birds	75%	1	45 s
sound	50%	1	30 s
duration	25%	1	15 s
	0%	1	0 s
	None	2	Few birds, no traffic
Traffic level	Low	4	Continuous/low traffic
	High	5	Intermittent/high traffic
	None	2	No traffic
Traffic type	Continuous	4	Continuous/low traffic
	Intermittent	5	Intermittent/high traffic
Trains	Present	5	Trains
Airplanes	Present	5	Airplanes, other sources

**Table 2:** The starting parameters P(n) (n=1,5) associated with each sound category to be used in Equation (1) (see also [38]).

*n*	P(*n*)	Identification
1	2	Many birds/species
2	1	Few birds/no traffic
3	0	No birds/no other sources
4	−1	Continuous/low traffic
5	−2	Intermittent/high traffic/other sources

**Table 3:** Quality intervals for the mean soundscape ranking index, 〈SRI〉.

Poor Quality	Medium Quality	Good Quality
SRI < 0	0 ≤ SRI ≤ 2	SRI > 2

**Table 4:** Summary of the main results for the exponent α in Equation (3), the parameters P(n) (n=1,5), the mean value of the SRI in the network, the dispersion of the SRI values σ (cf. Figure 5 and Figure 9), the mean value of dissimilarity $\langle D\rangle$ among all sites, the effective scale distance $R_{0}$ (cf. Equation (3)) and the total error of the fit Σ (Equation (4)).

	Initial Parameters	Optimized Parameters
α	1/3	1/2
*P*(1)	2.0	2.290
*P*(2)	1.0	0.766
*P*(3)	0.0	0.000
*P*(4)	−1.0	−1.528
*P*(5)	−2.0	−2.262
〈SRI〉	2.14	1.65
σ	1.54	1.88
$\langle D\rangle$	0.333	0.375
$R_{0}$	8500 m	2270 m
Σ	0.147	0.139

## Data Availability

Data are available upon request.
